# Advanced Stage Head and Neck Cancer Diagnosis: HEADSpAcE Consortium Health Systems Benchmarking Survey

**DOI:** 10.1002/hed.28094

**Published:** 2025-02-24

**Authors:** Grant Creaney, Mariél de Aquino Goulart, Alex McMahon, Claire Paterson, James McCaul, Sandra Perdomo, Laura Mendoza, Laia Alemany, Lidia Maria Rebolho Arantes, Paula Andrea Rodriguez Urrego, Tom Dudding, Mirana Pring, Marta Vilensky, Cecilia Cuffini, Silvia Adriana Lopez de Blanc, José Carlos de Oliveira, Shahid Pervez, Pierre Saintigny, Mauricio Cuello, Jaroslav Betka, Luis Felipe Ribeiro Pinto, Maria Paula Curado, Kazem Zendehdel, Lorenzo Richiardi, Maja Popovic, José Roberto de Podesta, Sandra Ventorin von Zeidler, Ricardo Mai Rocha, HEADSpAcE Consortium, R Adam, A Agudo, W Ahrens, L Alemany, S Alibhai, M Angel‐Pavon, N Anwar, L Arantes, P. E. Arantes, L Arguello, Y Avello, L Avondet, A. M. Baldión‐Elorza, C Batista‐Daniel, B Beraldi, B Berenstein, P Bernal, N Bernardino‐Rodrigues, J Betka, K Bilic‐Zimmermann, L Bouvard, M Botta, J Brenes, N Brenner, C Brentisci, C Burtica, M Cabañas, C Canova, E Cantor, G Carretero, A Carvalho, R Carvalho, L Chiusa, P Chopard, M Choulli, Q Chundriger, O Clavero, S Coelho‐Soares‐Lima, I Costa, M Cuello, C Cuffini, M. P. Curado, A. C. de Carvalho, J. C. de Oliveira, T Dias, T Dudding, E Duccini de Souza, I. C. Durant, E Ebrahimi, A Escallón, G. A. Fernandes, B Fervers, V Fiano, F Firme‐Figueira, R Furbino‐Villefort, V Gaborieau, R Gama, M Gangemi, P Garzino‐Demo, R Giglio, A Guasch, J Graça Sant’Anna, M Grega, A Gregório‐Có, M Hadji, J. A. Hakim, N Hayes, C. M. Healy, M Homero de Sá Santos, I Holcatova, K Hurley, M Insfran, G. C. Iorio, M Iqbaluddin Siddiqui, J Johannsen, M Kaňa, J. P. Klussmann, L. P. Kowalski, A Lagiou, P Lagiou, E Legal, J Lenzi, F Luiz Dias, Lyra González, W Machado‐Zorzaneli, G. J. Macfarlane, R Mai‐Rocha, M Maños, P Marinho‐de‐Abreu, J Mckay, C Mena, L Mendoza, E. F. Mendonça, L Meza, B Michels, M. S. Mineiro, C Moccia, P Mongelos, A. L. Montealegre‐Páez, F Morey‐Cortes, A Muñoz, A Ness, A Neves, M Oliva, H Ortiz, J Ortiz, M Osorio, V Ospina, O Ostellino, M Palau, S Paytubi‐Casabona, G Pecorari, S Perdomo, D. M. Pereira, O Pérol, S Pervez, A Pomata, J Polesel, M Popovic, A Poveda, C Prado, K Prager, M Pring, G Ramieri, H Rashidian, S Rasul, J Rego, R. M. Reis, H Renard, L. F. Ribeiro‐Pinto, U Ricardi, L Richiardi, G Riva, P Rodriguez‐Urrego, M Robinson, F Rodilla, I Rodriguez, M Rodríguez, P. E. Roux, T Saeed‐Ali, P Saintigny, J. J. Santivañez, C Scapultampo‐Neto, J Segovia, A Sena, M Sepideh, R Serrano, S. J. Sharma, O Siefer, S Smart, B. P. Sorroche, C Sosa, J. D. Souza, A Stura, S Thomas, S Thoms, O Torres, S Tous, G Ucross, A Valenzuela, J. R. Vasconcelos‐de‐Podestá, M Vilensky, S Von‐Zeidler, T Waterboer, A Whitmarsh, S Wright, K Zendehdel, A Znaor, Shaymaa Alwaheidi, Paul Brennan, Shama Virani, Al Ross, David I Conway

**Affiliations:** ^1^ School of Medicine, Dentistry and Nursing University of Glasgow Glasgow UK; ^2^ Glasgow Head and Neck Cancer (GLAHNC) Research Group Glasgow UK; ^3^ Beatson West of Scotland Cancer Centre Glasgow UK; ^4^ Queen Elizabeth University Hospital Glasgow UK; ^5^ International Agency for Research on Cancer (IARC) Lyon France; ^6^ Universidad Nacional de Asunción Asuncion Paraguay; ^7^ Institut Catala D'oncologia Barcelona Spain; ^8^ Hospital de Câncer de Barretos Barretos Brazil; ^9^ Department of Pathology and Laboratories Fundacion Santa Fe de Bogota Bogota Colombia; ^10^ Bristol Dental School University of Bristol Bristol UK; ^11^ Universidad De Buenos Aires Buenos Aires Argentina; ^12^ Virology Institute School of Medicine.Universidad Nacional de Córdoba Córdoba Argentina; ^13^ Facultad de Odontología Universidad Nacional de Córdoba Cordoba Argentina; ^14^ Araujo Jorge Hospital Brasil Goiânia Cancer Registry Goiania Brazil; ^15^ Aga Khan University Karachi Pakistan; ^16^ Centre Leon Berard Lyon France; ^17^ Service of Oncology Manuel Quintela Hospital Montevideo Uruguay; ^18^ Department of Otorhinolaryngology and Head and Neck Surgery, 1st. Faculty of Medicine Charles University in Prague, Faculty Hospital Motol Prague Czechia; ^19^ Brazilian Ministry of Health/Instituto National De Cancer Rio De Janeiro Brazil; ^20^ A. C. Camargo Centre Sao Paulo Brazil; ^21^ Tehran University of Medical Sciences Tehran Iran; ^22^ Cancer Epidemiology Unit, Department of Medical Sciences University of Turin, and CPO‐Piemonte Turin Italy; ^23^ Serviço de Cirurgia de Cabeça e Pescoço Associação Feminina de Educação e Combate ao Câncer Vitória Brazil; ^24^ Pathology Department Federal University of Espírito Santo Vitória Brazil; ^25^ School of Health, Education, Policing and Sciences Staffordshire University Stafford UK

**Keywords:** diagnostic pathway, head and neck cancer, health systems, stage at diagnosis

## Abstract

**Background:**

Globally, most people with head and neck cancers (HNCs) are diagnosed with advanced‐stage disease. HNC diagnostic stage has multifactorial explanations, with the role of health system factors not yet fully investigated.

**Methods:**

HNC centres (*n* = 18) from the HEADSpAcE Consortium were surveyed via a bespoke health system questionnaire covering a range of factors. Centres were compared using the least square means for the presence/absence of each health system factor to their proportion of advanced‐stage HNC.

**Results:**

Health system factors associated with lower proportion in advanced‐stage diagnosis were formal referral triaging (14%, 95% CI‐0.26, −0.03), routine monitoring of time from referral to diagnosis (16%, 95% CI‐0.27, −0.05), and fully publicly funded systems (17%, 95% CI‐0.29, −0.06). Several health systems factors had no routinely available data.

**Conclusions:**

Through identifying and monitoring health systems factors associated with lower proportions of advanced stage HNC, interventions could be developed, and systems redesigned, to improve early diagnosis.

**Abbreviations**
HEADSpAcE: Head and Neck Cancer in South America and Europe consortium
HIC: high income country
HNC: head and neck cancer
IARC: International Agency for Research on Cancer
ICBP: International Cancer Benchmarking Partnership
LMIC: low‐middle income country
WHO: World Health Organization

## 1 Background

Head and neck cancers (HNCs) comprising cancers of the oral cavity, pharynx, and larynx are the sixth most commonly diagnosed cancer group globally with 90% of HNCs being squamous cell carcinoma (SCC) [1]. Mortality rates are high [2], and extensive multi‐modal treatment is usually required, but often results in significant morbidities [3]. Stage at diagnosis influences treatment planning and is a key prognostic factor [4, 5], with advanced disease (Stage III and IV as per TNM 7th and 8th editions) [6, 7] resulting in poorer survival. Estimates from large international cohort studies have shown the proportion of advanced HNC to range from 54% in Europe [8] to 75% in South America [9]. Cancer registry analysis has shown that 59% of newly diagnosed HNCs in the United Kingdom were recorded as TNM stage III or IV in the national cancer registries in 2016–2018 [10]. Despite advances in understanding the causes and risks of developing HNC [2], preventative and early detection measures [11], and progress in treatments for HNCs including technological advances in radiotherapy and new immunotherapy regimens [12], there has been minimal improvements in survival from HNC observed in recent decades [1, 2].

Health systems are known to be complex with many challenges arising from dynamic interactions between patient factors, operational procedures, and organizational demands [13]. Currently, the literature investigating factors associated with diagnosis of advanced HNC does not include health system factors, and is limited to findings on individual race, type of health insurance, and is based in the United States of America [5, 14, 15], or is for the oral cavity subsite only where the main finding was the role of patient and professional awareness of oral cancer [16]. Recent studies investigating the role of health systems and diagnosis of cancer (but not including HNC), identified the potential role of technology, gatekeeping, finance, and centralisation of services on diagnostic pathways and patient experience toward diagnosis [15, 17, 18]. This study aims to explore the potential role for health system factors on the stage at diagnosis of HNC's exploring the different pathways to diagnosis across HEADSpAcE centres.

## 2 Methods

### 2.1 Study Design and Setting

The HEADSpAcE (Head and neck cancer in South America and Europe) Consortium is an international multicentre research programme coordinated by the International Agency for Research on Cancer—World Health Organization (IARC‐WHO) focused on investigating factors associated with advanced stage at diagnosis of HNCs including genomic, patient, socioeconomic, and health system factors [19]. The HEADSpAcE Consortium includes 18 HNC tertiary treatment centres: 10 from South America, six from Europe, and two from the Middle East (Table 1).

**TABLE 1:** HEADSpAcE head and neck cancer centres.

HEADSpAcE centre	Location	Country	Region	United Nations Human Development Index	World bank economy ranking	HNC cases diagnosed in 2019 (*n*)
Tehran University of Medical Sciences (TUMS)	Tehran	Iran	South Central Asia	High	Low‐middle income	56
Aga Khan University (AKU)	Karachi	Pakistan	South Central Asia	Low	Low‐middle income	602
Santa Fe de Bogotá Foundation University Hospital (FSFB)	Bogota	Colombia	South America	High	Low‐middle income	123
A.C. Camargo Cancer Centre (AC‐CCC)	Sao Paulo	Brazil	South America	High	Low‐middle income	823
Barretos Cancer Hospital (HCB)	Barretos	Brazil	South America	High	Low‐middle income	704
University of the Republic (UdelaR)	Montevideo	Uruguay	South America	Very high	High income country	110
National University of Cordoba (UNC)	Cordoba	Argentina	South America	very high	Low‐middle income	20
Hospital Santa Rita de Cassia—Women's Association of Education and Fight against Cancer (AFECC)	Vitoria	Brazil	South America	High	Low‐middle income	341
Goiânia Cancer Registry (GCR)	Goiânia	Brazil	South America	High	Low‐middle income	650
University of Buenos Aires (IOAR)	Buenos Aires	Argentina	South America	Very high	Low‐middle income	443
Brazilian National Cancer Institute (INCA)	Rio	Brazil	South America	High	Low‐middle income	1296
National University of Asuncion (NUA)	Asuncion	Paraguay	South America	High	Low‐middle income	155
Léon Bérard Center (CLB)	Lyon	France	Europe	very high	High income country	403
University of Bristol (UBRIS)	Bristol	UK	Europe	Very high	High income country	210
Catalan Institute of Oncology (ICO)	Barcelona	Spain	Europe	Very high	High income country	333
University of Turin (UNITO)	Turin	Italy	Europe	Very high	High income country	171
Charles University (CUNI)	Prague	Czechia	Europe	Very high	High income country	266
University of Glasgow (UGLA)	Glasgow	UK	Europe	Very high	High income country	543

This study utilized a systems survey approach through a self‐completed questionnaire, specifically designed for the centre leads of the HNC centres within the HEADSpAcE Consortium. Data collection were focussed on the health system in the year prior to the COVID‐19 pandemic (i.e., in 2019), questionnaires were returned between November 2020 and November 2022, with subsequent rounds of follow up with centres to check data quality and completeness. Each HEADSpAcE centre is linked in with local HNC clinical centres.

### 2.2 Data Sources and Measurement

A bespoke questionnaire was developed with reference to the literature [17] and in consultation with clinicians, healthcare managers, and administrators and collaborators from the HEADSpAcE Consortium. The questionnaire included both closed and open‐ended questions to assess the availability of data on health system domains and open‐ended questions to gather detailed information on the healthcare pathway to diagnosis; alongside data on the number of HNC cases diagnosed in 2019 and the proportion of these that were advanced‐stage at diagnosis. Additionally, local protocols or guidelines for referral and diagnosis of HNC where available were requested from each centre. Project leads in each of the 18 HNC centres were responsible for the completion of the questionnaire for their respective centre.

### 2.3 Health System Domains

Health system domains covered in the questionnaire included items on the availability and nature of: referral systems (assessing electronic pathways and triaging); quality/performance indicators (monitoring time from referral to diagnosis); diagnostic processes (centralized diagnoses, use of guidance); multidisciplinary teams (assessing comprehensiveness of care); technology (in relation to communication across the diagnostic system); financial models (evaluating funding structures); centre activity (measuring case‐load volume), and service structures (assessing degree of centralisation of services).

Following collation of the questionnaire responses; health system factors deriving from responses to each respective questionnaire domain with data available for benchmarking across all centres were identified (Table 2). Centres were categorized by the presence of the health system factor in their local HNC system (yes/no).

**TABLE 2:** Healthcare system questionnaire domains.

Health system domains	Topics in health system questionnaire	Benchmarking health system factor	Description of health system factor
Referral system	Referral guidance Referral volume Referral categories Referral methods and processes Triaging	Bespoke electronic Referral pathway	Bespoke electronic referral system used as main method of referral into specialist care
		Referral guidance	Guidance on referral processes and criteria is available for primary care/community care teams
		Triaging system	Formalized referral triaging/vetting of received referrals by specialist team
Quality/performance indicators	Waiting time from referral to diagnosis Waiting time from referral to first appointment Diagnostic investigation reporting time targets Routinely monitored performance indicators	Referral to diagnosis waiting time targets/monitored	Routinely monitored and reported from date of referral through to diagnosis date in entirety
Diagnostic processes	Diagnostic confirmatory procedures	Diagnosis made exclusively by HEADSpAcE centre	Diagnosis is usually only made at the HEADSpAcE centre for all patients and not at another service prior to referral
Multidisciplinary teams	Frequency of meetings Multidisciplinary composition of members	Comprehensive multi‐disciplinary team (MDT)	MDT includes representation from wide variety of specialists and health professionals and meets regularly
Technology	Communication methods Common electronic medical records	Common medical record	Shared record accessible by all health practitioners across primary care and secondary care
Workforce	Numbers/full‐time equivalent primary care clinicians in local system Numbers/full‐time equivalent specialist/secondary care in local system	Specialist HNC pathologists and radiologists	Both radiology and pathology specialists are available locally
Financial models	Additional patient costs Public/private/mixed/insurance	Fully publicly funded HNC centre	Fully Publicly funded HNC diagnosis and treatment, including dental checks
Centre activity	New cases diagnosed Source of referrals Proportion of advanced HNC	Large HNC patient volume	Centre treats ≥ mean number of cases per annum (402, from the 18 centres)
Service structure	Location of services “One‐stop” clinics	All diagnosis and treatment undertaken at one location in HEADSpAcE centre	All aspects of diagnosis and treatment happen in one hospital/physical site

### 2.4 Centre Health System Benchmarking Analysis

The centres were sorted by their proportion of advanced stage HNC diagnosed in 2019 (Table 3). Least square means tests were performed to calculate the absolute percentage difference and standard deviation in the proportion of advanced‐stage HNC for each health system factor (along with 95% confidence intervals and *p*‐values). Subgroup analyses and sensitivity analyses were also performed to ensure robustness of the findings (Table 4). All statistical analyses were performed using R software (R version 2022.02.2).

**TABLE 3:** Benchmarking proportion of advanced stage HNC against presence of health system factor.

Health System Factor	HEADSpAcE Centre																	
	CLB	UBRIS	ICO	FSFB	AC‐CCC	UNITO	TUMS	HCB	UdelaR	CUNI	UNC	UGLA	AKU	AFECC	GCR	IOAR	INCA	NUA
Estimated proportion of advanced stage HNC 2019	27%	38%	45%	46%	48%	50%	50%	50%	52%	55%	59%	65%	65%	69%	70%	76%	77%	90%
Electronic referral system for HNC	No	Yes	Yes	Yes	No	Yes	No	No	No	Yes	Yes	Yes	No	Yes	No	No	Yes	No
Common primary‐secondary care medical record	No	Yes	Yes	No	No	No	No	No	Yes	Yes	No	No	Yes	No	No	No	No	No
Higher patient volume (>Mean)	Yes	No	No	No	Yes	No	No	Yes	No	No	No	Yes	Yes	No	Yes	Yes	Yes	No
Single centralised site	Yes	No	Yes	Yes	No	No	Yes	No	Yes	Yes	No	No	No	No	No	Yes	Yes	Yes
Referral waiting times monitored	Yes	Yes	Yes	Yes	Yes	Yes	Yes	No	No	Yes	Yes	Yes	Yes	No	No	Yes	No	No
Initial diagnosis made exclusively at centre	No	Yes	Yes	No	No	Yes	No	No	Yes	Yes	No	Yes	Yes	No	No	No	No	No
Comprehensive MDT	Yes	Yes	Yes	Yes	Yes	Yes	No	No	No	Yes	Yes	Yes	No	Yes	No	Yes	Yes	No
Specialist HNC radiologists and pathologists	No	Yes	Yes	Yes	Yes	Yes	Yes	Yes	No	Yes	Yes	Yes	Yes	No	Yes	Yes	No	No
Referrals formally triaged	Yes	Yes	Yes	Yes	No	Yes	Yes	Yes	Yes	Yes	Yes	Yes	No	Yes	Yes	Yes	No	No
Referral guidance	No	Yes	Yes	Yes	No	Yes	No	No	No	Yes	No	Yes	No	Yes	No	No	No	No
Exclusively publicly funded system	Yes	Yes	Yes	No	No	Yes	No	No	Yes	Yes	No	Yes	No	No	No	No	No	No

**TABLE 4:** Least square means analysis of healthcare system factors.

Health system factor	Y/N	Mean proportion advanced stage HNC 2019 (SD)	Difference in means (95% Cis)	*p*
HNC electronic referral system	N	0.59 (0.19)	—	—
	Y	0.56 (0.13)	−0.03 (−0.18, 0.13)	0.7023
Common primary/secondary medical record	N	0.60 (0.17)	—	—
	Y	0.51 (0.10)	−0.10 (−0.23, 0.03)	0.1221
Higher patient volume (> mean)	N	0.55 (0.15)	—	—
	Y	0.60 (0.17)	0.02 (−0.13, 0.16)	0.8111
Single site/location	N	0.57 (0.11)	—	—
	Y	0.58 (0.20)	0.00 (−0.19, 0.19)	0.9584
Routine monitoring of referral waiting times	N	0.68 (0.15)	—	—
	Y	0.52 (0.13)	−0.16 (−0.27, −0.05)	0.0069
Initial diagnosis within centre	N	0.60 (0.18)	—	—
	Y	0.53 (0.10)	−0.08 (−0.21, 0.06)	0.2357
Comprehensive MDT	N	0.63 (0.16)	—	—
	Y	0.55 (0.15)	−0.08 (−0.20, 0.04)	0.1564
Specialist HNC pathologists and radiologists	N	0.63 (0.24)	—	—
	Y	0.55 (0.11)	−0.07 (−0.24, 0.11)	0.4319
Referral triaging system	N	0.70 (0.18)	—	—
	Y	0.54 (0.13)	−0.14 (−0.26, −0.03)	0.0179
Referral guidance	N	0.60 (0.18)	—	—
	Y	0.53 (0.11)	−0.08 (−0.22, −0.06)	0.2458
Entirely publicly funded patient finance	N	0.64 (0.14)	—	—
	Y	0.47 (0.12)	−0.17 (−0.29, −0.06)	0.008

### 2.5 HNC Diagnostic Pathway Description and Harmonization

Open questions were included in the questionnaire which asked for descriptions of each centre's pathway to HNC diagnosis. These responses were clarified with follow up online discussions with centre leads where required. The interval approach that forms the Aarhus pathway for cancer research [20, 21, 22] was used as a framework to collate and harmonize the range of diagnostic pathways across centres for all HNC subsites with the aid of Lucidchart (lucid.co) digital mapping software.

## 3 Results

### 3.1 Centre Health System Benchmarking

The centres were ranked and benchmarked by the proportion of advanced stage HNC in 2019 which ranged from 27% (CLB, Lyon, France) to 90% (NUA, Asunción, Paraguay), along with the presence or absence of health system factors (Table 3). Nine out of 18 centres have electronic referral systems, while only five have integrated common primary‐secondary care medical records. Eight centres manage a higher number of patients per year than the mean (mean *n* = 402, range 20–1296), and nine operate from a single centralized site. Monitoring referral to diagnosis waiting times is undertaken in 12 centres. Eleven centres employ comprehensive multidisciplinary teams, and 12 have specialist HNC radiologists and pathologists. Formal triage of referrals occurs in 13 centres, but only seven have formal referral guidance. There were diverse funding models for the health systems with seven exclusively publicly funded centres. No centre exhibited all of the health system factors assessed and all centres had at least one of the assessed health system factor present.

The presence of several health systems factors within the HNC centres included in this analysis were associated with a lower proportion of advanced stage HNC (Table 4). Of all the factors analyzed, three were strongly associated with a lower proportion of advanced stage HNC diagnoses when they were part of a centre's HNC system: (i) routine monitoring of waiting times from referral to diagnosis had a 16% lower proportion in advanced stage HNC (95% confidence interval (CI) −0.27, −0.05; *p*‐value 0.007); (ii) having a formal referral triaging process showed a 14% lower proportion (95% CI −0.26, −0.03; *p*‐value 0.0179); and (iii) centres with a publicly funded patient finance model/universal health coverage had a 17% lower proportion (95% CI −0.29, −0.06; *p*‐value 0.008). Centres with higher patient volume (2% (95% CI −0.13, 0.16)) and centralisation of centre services (*% (95% CI −0.19, 0.19)) showed no evidence of a higher proportion of advanced stage HNC while the remaining health system factors showed no evidence of association with HNC stage at diagnosis.

Overall, centres in HICs (mean = 47%) had a 17% lower proportion of advanced‐stage HNC than centres in LMICs (mean = 64%) (95% CI −0.30, −0.03 *p*‐value 0.022).

Several of the domains included in the questionnaire were found to have no routinely available data across HEADSpAcE centres, meaning that several gaps in health system factors were identified (Table 5). This may be important to contextualize the results of this study and aid discussion on the potential role of health systems factors and HNC diagnosis. For example, while the stage at diagnosis is recorded individually for each patient, it is not routinely aggregated and reported/monitored as a management system measure; there was also no formal routinely reported data on the source of suspected cancer referrals in any centre. Additionally, data on workforce composition and availability in both primary and secondary care is not routinely available. Quality Performance Indicators (QPIs) in HNC were primarily focussed on post‐diagnostic events and treatment. In addition, total diagnostic time (from referral to diagnosis) is not commonly reported; instead, centres more commonly report sub‐time points such as the first appointment at the HNC specialist centre and treatment initiation.

**TABLE 5:** Gaps in healthcare system questionnaire responses.

Health system domain	Missing health system factor	Explanation of missing Data
Centre activity	Proportion of advanced stage HNC	Stage at Diagnosis recorded individually for each patient but not routinely reported as a system measure
	“One‐stop” clinics	Only present in two centres and only for some subsites
	Source of suspected cancer referrals	No formal routinely reported data on referral source
Workforce	Number of primary care and specialist clinicians in local system	Data on workforce composition and availability is not formally routinely available
Referral system	Proportion of suspected cancer referrals with confirmed HNC diagnosis	Often audited but not formally routinely reported
	Guidelines for referral processes	Guidance for diagnostic procedures near universally available but seldom for referral
Quality performance indicators	QPIs relating to referral/pre‐diagnosis	Few or none across centres, QPIs largely focussed on treatment/post‐diagnosis events
	Referral waiting times	Total diagnostic time not commonly reported: sub‐time points such as first appointment and time to treatment more commonly used
	Targets/waiting times reporting for specialist investigations	Not formally reported in most centres

### 3.2 Harmonized HNC Diagnostic Pathway

**FIGURE 1:**
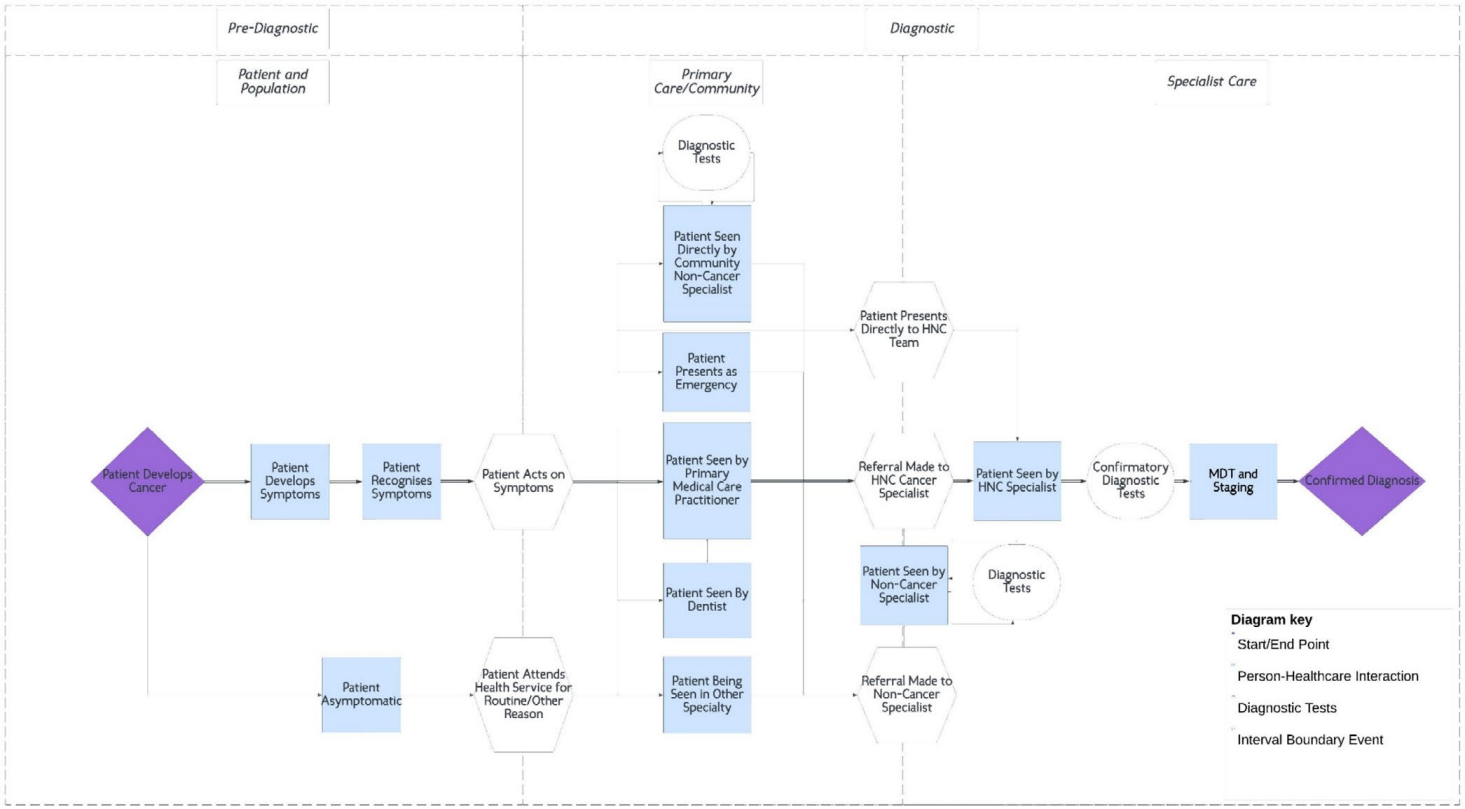
HEADSpAcE HNC diagnostic pathway. [Color figure can be viewed at wileyonlinelibrary.com]

A simple HNC diagnostic pathway was harmonized from the HEADSpAcE HNC centre pathways to capture and collate the variation in diagnostic pathways across all HNC centres in the HEADSpAcE consortium (Figure 1). This pathway defines the various routes through which people are diagnosed with HNC, including direct presentation to specialist hospital services and acute presentations to emergency departments. The dominant pathway across centres was that of a hybrid model where diagnosis is made either at the centre itself or within primary care/community care before being referred on, which was more prominent in South American centres (*n* = 10), with other centres having a specialist‐only diagnostic model with patients presenting to primary/community health services and subsequently being referred to a hospital specialist for further investigation and diagnosis (*n* = 8). Routes through primary care were split between patients who went to dental services and those who went to medical services. This pathway also captures the potentially varying routes that a patient might traverse to getting a diagnosis of HNC depending on the cancer subsite with some OCCs being initially detected by dental clinicians. This HEADSpAcE Head and Neck Cancer Diagnostic Pathway provides a formalized description of the contextual work system (“work as done” [23]) to capture the heterogeneity of pathways from the international health systems included in the consortium.

## 4 Discussion

This study explored for the first time the role of health system factors associated with the diagnosis of advanced stage HNC across various international centres, encompassing both low/middle‐income and high‐income countries. Key findings indicate that three health system factors are associated with a lower proportion of advanced stage HNC diagnoses: routine monitoring of waiting times from referral to diagnosis, having a formal referral triaging process, and being fully publicly funded.

The findings align with previous research that highlights the importance of accessible and efficient healthcare systems in improving cancer outcomes [24, 25, 26, 27]. The significant lower proportions in advanced stage HNC associated with monitoring waiting times, formal referral triaging, and publicly funded health systems underscore the potential benefits of these practices. However, the lack of impact from service centralisation and higher patient volumes suggests that these factors may not be as crucial in the context of HNC diagnosis. The impact of centralisation of services, which is closely linked with higher numbers of cases, has previously been found to be associated with better survival outcomes in esophageal cancers at a regional level [28] but it is not clear whether this is through improved diagnostic pathways or in relation to other treatment/care services. An analysis of national trends in breast and ovarian cancers in France found centralisation to be associated with increased quality of care but increased inequalities in access to care [29]. A modeling of centralisation of specialist cancer services for rectal cancer in the UK at the national level showed the potential travel impacts on patients but showed limited impact on stage at diagnosis [30].

While there was a 17% lower proportion of advanced‐stage HNC in centres located within HICs when compared to LMICs, care should be taken when interpreting this result as some centres as there was wide variation—for example within HIC, UoG in Glasgow, Scotland (65%), had a higher proportion of advanced‐stage HNC than those in LMICS, such as those in AC‐CCC, Sao Paulo, Brazil (48%). These differences could also relate to within country inequalities and the socioeconomic profile of people with HNC and other determinants of advanced stage disease at the centre level [31].

One of the key data gaps identified was the lack of routinely reported data on the proportion of advanced stage at diagnosis within each centre. This measure was not readily available or used in management/service monitoring. Centre proportion of advanced stage HNC had to either be calculated or clinically estimated from clinical records/lists. This was surprising given the important relationship of stage at diagnosis in determining treatment (service) planning and in prognosis [8, 17, 32, 33, 34]. Similarly, stage of HNC is not a routinely reported measure in cancer registries. This has only recently been captured in the UK with analyses showing that 59% of HNC cases are diagnosed as stage III or IV [10] which puts the UK target of 75% of cancers being diagnosed at stage I or II by 2028 very unlikely to be achieved for HNC [35].

Additional important findings in this study were health system domains in which data were unavailable from any centre, these included routine information on workforce and source of referral. These domains had previously been identified as potential health systems factors in cancer diagnosis [17, 36]. The lack of these data highlight gaps in monitoring of the diagnostic pathway in all centres which could be utilized for health system quality improvement. These variations in structural and operational characteristics could impact the quality and efficiency of care.

The focus of research to date in health systems factors in cancer diagnosis has mainly been on other cancer groups such as breast, lung, and colon cancers [37, 38, 39, 40, 41]. These studies were a primary source of the health system domains that informed the questionnaire developed for this study. This prior research focus may be reflective of the higher disease burden and more ready availability of high‐quality reported data for these cancer groups historically [42].

Our newly devised HEADSpAcE HNC Diagnostic Pathway, synthesized from the consortium centres' individual pathways to diagnosis, offers a novel lens through which future HNC research and intervention development can be undertaken. It provides a real‐world framework that is likely to cover the majority of patients' diagnostic journeys and can aid in planning and evaluation of interventions aiming to address variation and inequalities in the pathway.

To our knowledge, this is the first study addressing centre‐level health system factors on stage at diagnosis in HNC, and the results are strengthened by inclusion of data from a number of HNC systems from across the world. The centres included in this study were heterogeneous in their geography, healthcare system structure, World Bank economic ranking, and United Nations Human Development Index, allowing for a broader analysis of health system factors, but my not reflect the total range of HNC systems internationally.

This study has a number of limitations. As noted by Brown et al. [17] in their narrative review, attributing causality to an outcome due to any particular health system factor is challenging due to the significant complexity and socio‐organizational environment in which healthcare systems exist. Our study has only begun to explore the potential influence these factors but had limited access to wider socioeconomic system data, however, further triangulation with other data and ongoing approaches within the HEADSpAcE consortium including analysis of prospective individual patient HNC cohort and qualitative centre case‐studies (IARC) will enhance the literature on this subject. Similarly, not considering health/cancer policy related information is another limitation. The International Cancer Benchmarking Partnership (ICBP), which does not include HNC, has shown that policy has a crucial role to play in cancer outcomes [43]. The wider cancer/public health system could be defined as starting with the self‐detection of a health problem and subsequent health seeking element of a patient's interaction with services was not fully captured here [44]. This could include screening services/activities, although, there is limited current evidence for formal screening programmes for HNC [45, 46] and improvements in early detection of HNC may have come from opportunistic screening, for example in primary care dental services [46], and in better joined up primary and secondary/tertiary services and care pathways.

This study considers only the cancer system to the point of diagnosis, but investigation of the role of health system factors in HNC treatment and survivorship should also be a priority for future research in order to have a comprehensive whole‐system approach to reducing the devastating burden of HNC.

## 5 Conclusions

This study reveals the role that health system factors play in the burden of advanced stage HNC diagnosed; with processes that monitor referral to diagnosis waiting times and formally triage referrals, along with systems within a fully publicly funded model being associated with lower centre‐level proportion of advanced HNC.

It is key that in order to shift the burden of disease from advanced to early stage, more attention should be given to routinely monitoring the burden of advanced disease. A diagnostic pathway for HNC has been proposed to allow better planning for future development of interventions or health system/policy change or innovation to improve the diagnostic care pathway for HNC.

## Author Contributions

**G.C.:** data collection, collation, conceptualisation, analysis, primary manuscript draft and revisions, qualitative analysis. **D.C.** and **A.R.:** conceptualisation, manuscript reviews. **M.G.** and **A.M.:** analysis support, manuscript reviews. All authors reviewed, and approved manuscript.

## Disclosure

Where authors are identified as personnel of the International Agency for Research on Cancer/World Health Organization, the authors alone are responsible for the views expressed in this article and they do not necessarily represent the decisions, policy or views of the International Agency for Research on Cancer/World Health Organization.

## Ethics Statement

The authors have nothing to report.

## Consent

The authors have nothing to report.

## Conflicts of Interest

The authors declare no conflicts of interest.

## Data Availability

Data used for analysis are provided in the paper, qualitative data may be available on specific request to the main author.

## Appendix A

HEADSpAcE Consortium Group: Adam R^28^, Agudo A^3^, Ahrens W^38^, Alemany L^3^, Alibhai S^13^, Angel‐Pavon M^3^, Anwar N^31^, Arantes L^4^, Arantes PE^7^, Arguello L^26^, Avello Y^16^, Avondet L^30^, Baldión‐Elorza AM^16^, Batista‐Daniel C^32^, Beraldi B^19^, Berenstein B^20^, Bernal P^16^, Bernardino‐Rodrigues N^32^, Betka J^10^, Bilic‐Zimmermann K^3^, Bouvard L^1^, Botta M^7^, Brenes J^3^, Brenner N^21^, Brentisci C^29^, Burtica C^16^, Cabañas M^33^, Canova C^41^, Cantor E^16^, Carretero G^3^, Carvalho A^23^, Carvalho R^4^, Chiusa L^29^, Chopard P^1^, Choulli M^3^, Chundriger Q^13^, Clavero O^3^, Coelho‐Soares‐Lima S^14^, Costa I^14^, Cuello M^5^, Cuffini C^44^, Curado MP^7^, de Carvalho AC^1^, deOliveira JC^45^, Dias T^4^, Dudding T^8^, Duccini de Souza E^19^, Durant IC^22^, Ebrahimi E^1^, Escallón A^16^, Fernandes GA^7^, Fervers B^17^, Fiano V^15^, Firme‐Figueira F^32^, Furbino‐Villefort R^32^, Gaborieau V^1^, Gama R^23^, Gangemi M^29^, Garzino‐Demo P^27^, Giglio R^20^, Guasch A^3^, Graça Sant'Anna J^32^, Grega M^25^, Gregório‐Có A^32^, Hadji M^24^, Hakim JA^16^, Hayes N^9^, Healy CM^39^, Homero de Sá Santos M^19^, Holcatova I^10^, Hurley K^8^, Insfran M^11^, Iorio GC^28^, Iqbaluddin Siddiqui M^13^, Johannsen J^18^, Kaňa M^25^, Klussmann JP^18^, Kowalski LP^22^, Lagiou A^35^, Lagiou P^36^, Legal E^11^, Lenzi J^19^, Luiz Dias F^14^, Lyra González^5^, Machado‐Zorzaneli W^32^, Macfarlane GJ^42^, Mai‐Rocha R^19^, Maños M^3^, Marinho‐de‐Abreu P^32^, McKay J^1^, Mena C^11^, Mendoza L^11^, Mendonça EF^12^, Meza L^11^, Michels B^21^, Mineiro MS^12^, Moccia C^15^, Mongelos P^11^, Montealegre‐Páez AL^43^, Morey‐Cortes F3, Muñoz A^16^, Ness A^8^, Neves A^7^, Oliva M^3^, Ortiz H^33^, Ortiz J^11^, Osorio M^11^, Ospina V^16^, Ostellino O^29^, Palau M^16^, Paytubi‐Casabona S^3^, Pecorari G^27^, Perdomo S^1^, Pereira DM^20^, Pérol O^17^, Pervez S^13^, Pomata A^33^, Polesel J^37^, Popovic M^15^, Poveda A^43^, Prado C7, Prager K^21^, Pring M^8^, Ramieri G^27^, Rashidian H^24^, Rasul S^13^, Rego J^19^, Reis RM^4^, Renard H^1^, Ribeiro‐Pinto LF^14^, Ricardi U^28^, Richiardi L^15^, Riva G^27^, Rodriguez‐Urrego P^16^, Robinson M^40^, Rodilla F3, Rodriguez I^11^, Rodríguez M^11^, Roux PE^17^, Saeed‐Ali T^13^, Saintigny P^17^, Santivañez JJ^16^, Scapultampo‐Neto C^4^, Segovia J^16^, Sena A^19^, Sepideh M^24^, Serrano R^11^, Sharma SJ^18^, Siefer O^18^, Smart S^2^, Sorroche BP^4^, Sosa C^33^, Souza JD^7^, Stura A^29^, Thomas S^8^, Thoms S^8^, Torres O^16^, Tous S^3^, Ucross G^16^, Valenzuela A^11^, Vasconcelos‐de‐Podestá JR^19^, Vilensky M^20^, von‐Zeidler S^32^, Waterboer T^21^, Whitmarsh A^8^, Wright S^2^, Zendehdel K^24^, Znaor A^1^.

1. Genomic Epidemiology Branch, International Agency for Research on Cancer (IARC/WHO), Lyon, France.

2. University of Glasgow, Glasgow, United Kingdom.

3. Catalan Institute of Oncology (ICO), Barcelona, Spain.

4. Molecular Oncology Research Center, Barretos Cancer Hospital, Barretos, Sao Paolo, Brazil.

5. Servicio de Oncología Clínica Hospital de Clínicas, Universidad de la República, Montevideo, Uruguay.

7. Group of Epidemiology and Statistics on Cancer, A.C. Camargo Cancer Center, Sao Paolo, Brazil.

8. Bristol Dental School, University of Bristol, Bristol, United Kingdom.

9. UTHSC Center for Cancer Research, University of Tennessee Health Science Institute, Memphis, United States.

10. Charles University, Prague, Czech Republic.

11. Institute for Health Sciences Research, National University of Asunción (UNA), San Lorenzo, Paraguay.

12. Hospital Câncer Araújo Jorge, Goiania, Goiás, Brazil.

13. Aga Khan University Hospital, Karachi, Pakistan.

14. Brazilian National Cancer Institute (INCA), Rio de Janeiro, Brazil.

15. Department of Medical Sciences, Cancer Epidemiology Unit, University of Turin, Turin, Italy.

16. SantaFe de Bogotá Foundation University Hospital, Bogotá, Colombia.

17. Léon Bérard Center, Lyon, France.

18. University of Cologne, Cologne, Germany.

19. Women's Association for Cancer Education and Control Hospital Santa Rita de Cassia, Vitoria, Brazil.

20. Institute of Oncology Angel H. Roffo, University of Buenos Aires, Argentina.

21. Division of Infections and Cancer Epidemiology, German Cancer Research Center (DKFZ).

22. Head and Neck Surgery and Otorhinolaryngology Department, Oncology Surgery, A.C. Camargo Cancer Center, Sao Paolo, Brazil.

23. Department of Head and Neck Surgery, Barretos Cancer Hospital, Barretos, Sao Paolo, Brazil.

24. Cancer Research Center, Tehran University of Medical Sciences, Teran, Iran.

25. University Hospital in Motol, Prague, Czech Republic.

26. National Instiute of Cancer, National Cancer Institute, Ministry of Public Health and Social Welfare, Capiatá, Paraguay.

27. Department of Surgical Sciences, University of Turin, Turin, Brazil.

28. Department of Oncology, University of Turin, Turin, Brazil.

29. AOU City of Health and Science of Turin, Turin, Brazil.

30. University of Buenos Aires, Argentina.

31. Faculty of Science and Technology, University of Central Punjab.

32. Federal University of Espírito Santo, Vitoria, Brazil.

33. National Cancer Institute, Ministry of Health, Capiatá, Asunción, Paraguay.

34. Beatson West of Scotland Cancer Centre, NHS Greater Glasgow and Clyde, Glasgow, UK.

35. School of Public Health, University of West Attica, Athens, Greece (GR).

36. School of Medicine, National and Kapodistrian University of Athens, Greece (GR).

37. National Cancer Institute, IRCCS (IT).

38. Leibniz Institute for Prevention Research and Epidemiology—BIPS, Bremen, Germany.

39. Trinity College School of Dental Science Dublin, Ireland.

40. Department of Cellular Pathology, Royal Victoria Infirmary, UK.

41. University of Padova (IT), Padova, Italy.

42. MRC Centre for Musculoskeletal Health and Work, University of Aberdeen, Scotland, UK.

43. Faculty of Medicine, El Bosque University, Bogotá, Colombia.

44. National University of Córdoba, Córdoba, Argentina.
